# Enhanced nucleotide excision repair capacity in lung cancer cells by preconditioning with DNA-damaging agents

**DOI:** 10.18632/oncotarget.4610

**Published:** 2015-07-20

**Authors:** Ji Ye Choi, Jeong-Min Park, Joo Mi Yi, Sun-Hee Leem, Tae-Hong Kang

**Affiliations:** ^1^ Department of Biological Science, Dong-A University, Busan, Korea; ^2^ Research Center, Dongnam Institute of Radiological & Medical Sciences, Busan, Korea

**Keywords:** nucleotide excision repair, XPA, SIRT1, chemoresistance

## Abstract

The capacity of tumor cells for nucleotide excision repair (NER) is a major determinant of the efficacy of and resistance to DNA-damaging chemotherapeutics, such as cisplatin. Here, we demonstrate that using lesion-specific monoclonal antibodies, NER capacity is enhanced in human lung cancer cells after preconditioning with DNA-damaging agents. Preconditioning of cells with a nonlethal dose of UV radiation facilitated the kinetics of subsequent cisplatin repair and vice versa. Dual-incision assay confirmed that the enhanced NER capacity was sustained for 2 days. Checkpoint activation by ATR kinase and expression of NER factors were not altered significantly by the preconditioning, whereas association of XPA, the rate-limiting factor in NER, with chromatin was accelerated. In preconditioned cells, SIRT1 expression was increased, and this resulted in a decrease in acetylated XPA. Inhibition of SIRT1 abrogated the preconditioning-induced predominant XPA binding to DNA lesions. Taking these data together, we conclude that upregulated NER capacity in preconditioned lung cancer cells is caused partly by an increased level of SIRT1, which modulates XPA sensitivity to DNA damage. This study provides some insights into the molecular mechanism of chemoresistance through acquisition of enhanced DNA repair capacity in cancer cells.

## INTRODUCTION

Effective treatment of cancers usually requires the use of genotoxic chemotherapy. In most cases, multiple drugs are used, as resistance to single agents occurs almost universally. However, this causes many side effects for patients and thus, elucidation of mechanisms that confer chemoresistance has been a major goal of cancer biologists for decades. Cisplatin is one of the most commonly prescribed chemotherapeutic agents and produces platinum-DNA adducts, including intra- and inter-strand adducts and DNA-protein crosslinks [1, 2]. In humans, these lesions are removed primarily by nucleotide excision repair (NER) and hence, the status of NER is a critical indicator of the success of chemotherapy with cisplatin [3–5]. The importance of NER is highlighted by the finding that defects in this pathway result in hypersensitivity to cisplatin, and that restoration of NER activity reduces sensitivity to more normal levels [6, 7]. In spite of the critical role of NER in cisplatin resistance in cancer cells, however, the change in NER kinetics upon sequential cisplatin treatment or upon cisplatin treatment followed by treatment with another type of NER-dependent DNA-damaging agent, such as UV radiation, has not been precisely investigated due to the limitation of analytical tools such as cisplatin-DNA adduct-specific monoclonal antibody.

NER is a complex process carried out by seven xeroderma pigmentosum (XP) proteins (XPA to XPG) and approximately two dozen non-XP proteins [8]. Importantly, upregulation of only a few rate-limiting components of the NER system is necessary to increase a cell's capacity for NER. One such important rate-limiting factor is XPA, which has been found to be overexpressed in cisplatin-resistant cancers [9, 10]. In ovarian cancer, XPA was shown to be expressed at a higher level in tumors of patients resistant to cisplatin treatment [11]. Low levels of XPA are expressed in testicular cancer, which is generally very responsive to cisplatin, providing further correlative evidence for the importance of NER in cisplatin resistance [12, 13]. The rate-limiting effect of XPA in NER has been demonstrated in normal human fibroblasts (NHFs) as well. When XPA was downregulated to 60%, 10%, and 4% of its original value in the NHF-1 cell line in a controlled manner by titrating the amount of *XPA* siRNA, the rates of NER of both the 6-4 photoproduct (6-4PP) and the cyclobutane pyrimidine dimer (CPD), the two major types of DNA lesion caused by UV irradiation, were proportionally reduced [14]. Although the rate of repair of CPDs was linearly correlated with the level of XPA, the rate of 6-4PP repair exhibited a parabolic relationship with the XPA level, which is consistent with the well-established fact that the 6-4PP is repaired at a 5–10-fold faster rate than the CPD [15].

The steady-state level of XPA is mainly controlled by the circadian clock [16, 17], HERC2 [18, 19] and SIRT1 [20, 21]. The transcriptional activity of the circadian clock induces a daily rhythm of XPA gene expression, whereas HERC2 functions as an E3 ubiquitin ligase for XPA degradation in a proteasome-dependent fashion. The half-life of XPA protein is approximately 4 h in the absence of DNA damage, but much longer in the presence of DNA damage [19]. In response to DNA damage, XPA is phosphorylated by ATR kinase, which stabilizes XPA by preventing its association with HERC2 [18]. Thus, ATR activity in response to DNA damage can be utilized to a certain extent as a surrogate marker for NER activity. SIRT1, a NAD^+^-dependent histone deacetylase, also plays a critical role in the NER pathway. A recent study revealed that SIRT1 can deacetylate XPA and that this is required for interaction with replication protein A (RPA) and optimal NER activity [20].

Because of the significance of the NER capacity in a mechanism of chemoresistance in cancer cells, we decided to investigate the change in NER capacity after preconditioning of cells with a nonlethal dose of a DNA-damaging agent, which generates cells that mimic resistant cells after chemotherapy. For NER kinetic analysis we employed lesion-specific monoclonal antibodies to detect UV-induced CPD or 6-4PP and cisplatin-induced platinum-GpG adduct [22]. We found that the preconditioning renders cancer cells more resistant to a subsequent lethal dose of DNA-damaging agent by modulating the sensitivity of XPA association with DNA lesions hence, enhancing NER activity and conferring chemoresistance on cancer cells.

## RESULTS

**Figure 1:**
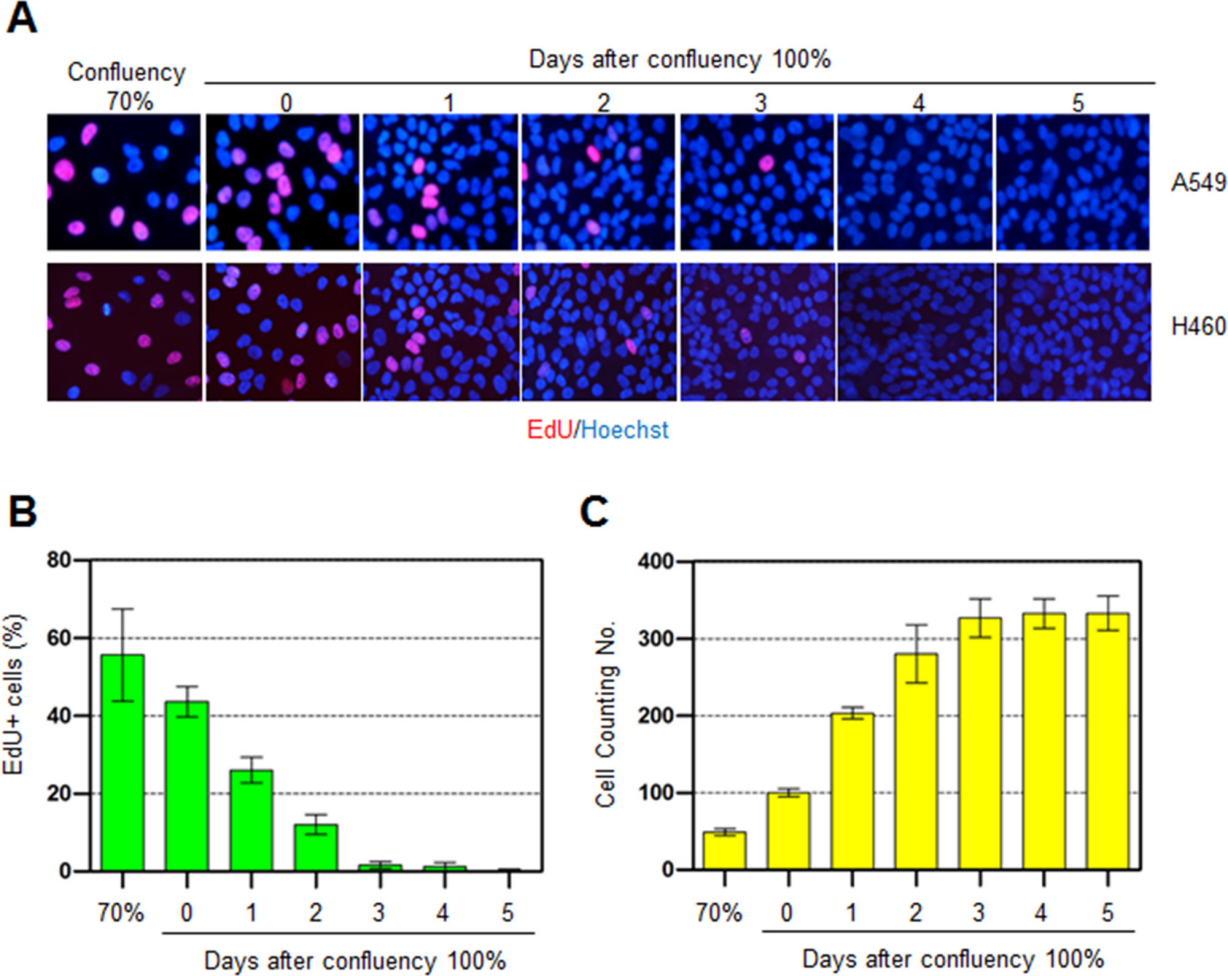

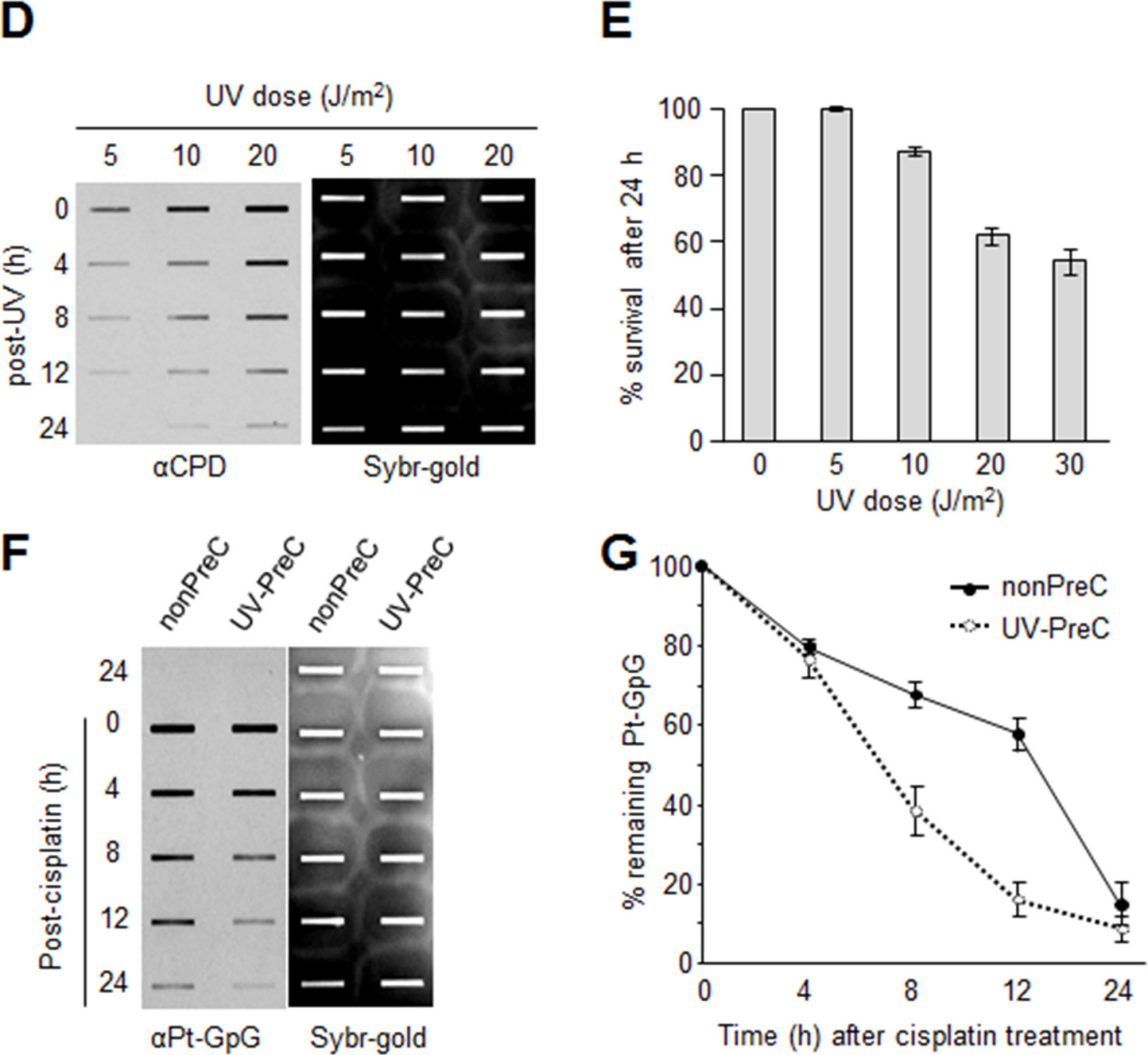
Preconditioning of cells with UV irradiation facilitates subsequent repair of cisplatin-induced damage **A.** A549 and H460 lung carcinoma cells were grown to the indicated density 70% or 100%. The 100% confluency is designated at the time when there is no space among the cells. EdU was added 2 h before fixation at the indicated culture density and days after 100% confluent. **B.** EdU-positive A549 cell numbers were counted among 1000 cells. **C.** The number of A549 cells in at day 0 of 100% confluent was designated as 100 control. The cell numbers from the other samples were plotted as relative values compared to control. The bars and error bars represent the mean ± s.d (*n* = 3). **D.** A549 cells irradiated with the indicated UV doses were allowed to carry out repair for the indicated times, followed by isolation of genomic DNA and immunoslot blotting analysis to detect residual cyclobutane pyrimidine dimers (CPDs). After immunoslot blotting, the membrane was counterstained with SYBR-Gold for a loading control of genomic DNA. **E.** Cell viability after UV irradiation was assessed by fluorescence-based cell viability assay. Constitutive protease activity within live cells was measured using a fluorogenic and cell permeable peptide substrate using CellTiter-Fluor Cell Viability Assay kit. The fluorescent signal obtained from mock-treated cells was designated as 100 and the relative values obtained from UV-exposed cells were plotted. The bars and error bars represent the mean ± s.d (*n* = 3). **F.** Removal rates of platinum-GpG (Pt-GpG) from nonpreconditioned (nonPreC) or UV-preconditioned (UV-PreC) cells. Cells were either mock-treated (nonPreC) or 5 J/m^2^ of UV-treated (UV-PreC) and kept for 24 h and followed by 10 μM of cisplatin treatment for 2 h and then culture medium was changed to wash out residual cisplatin in the medium. Recovery times were allowed for the indicated times and genomic DNAs obtained from each time point were assessed by immunoslot blotting using Pt-GpG-specific monoclonal antibody. **G.** The quantitative analysis for (F). The bars and error bars represent the mean ± s.d from three independent experiments.

**Figure 2:**
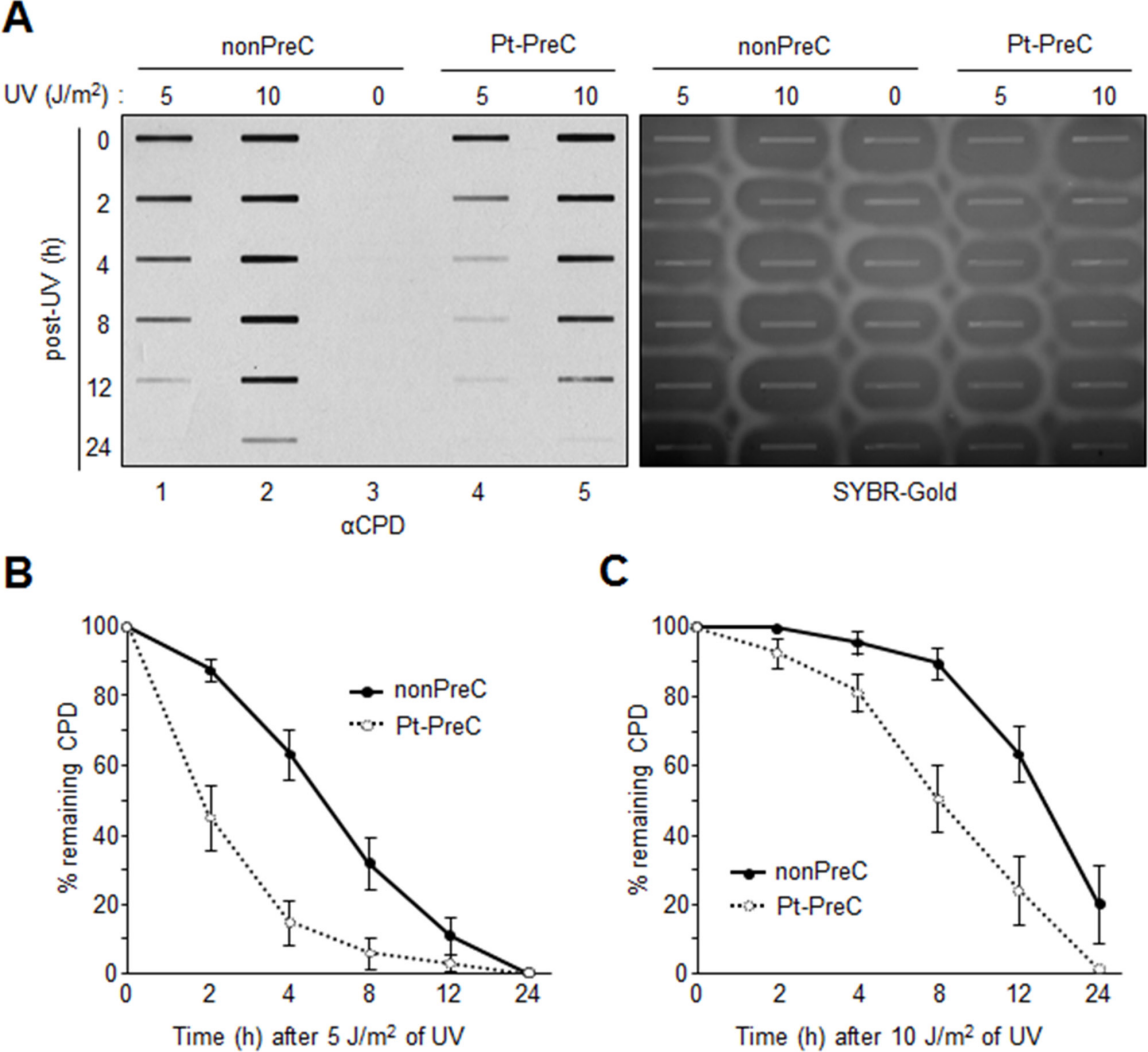
Enhanced UV-induced CPD repair activity following Pt-PreC **A.** The residual CPDs in genomic DNA of nonpreconditioned cells (nonPreC) or cells preconditioned with 5 μM of cisplatin (Pt-PreC) were assessed by immunoslot blotting. After immunoslot blotting the membrane was counterstained with SYBR-Gold for a loading control of genomic DNA. **B.** 5 J/m^2^ or **C.** 10 J/m^2^ of UV- induced CPD repair kinetics were measured from cells conditioned with nonPreC or Pt-PreC.

**Figure 3:**
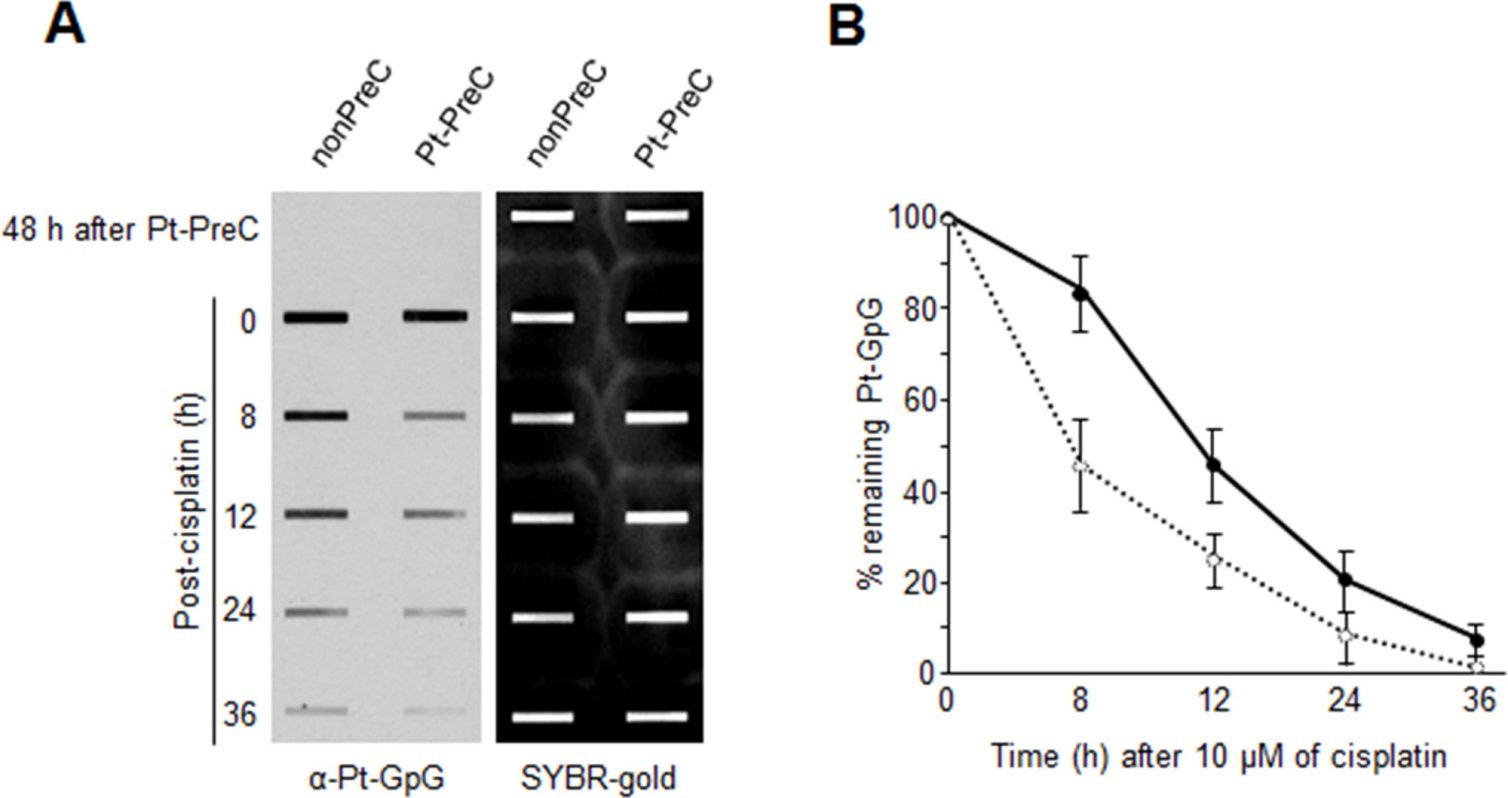
Enhanced Pt-GpG adduct removal by Pt-PreC **A.** Cells preconditioned with 5 μM of cisplatin or mock were treated with 10 μM of cisplatin, and Pt-GpG adduct removal rate was measured using immunoslot blotting with Pt-GpG adduct-specific monoclonal antibody. **B.** The quantitative analysis for (A) The bars and error bars represent the mean ± s.d from three independent experiments.

**Figure 4:**
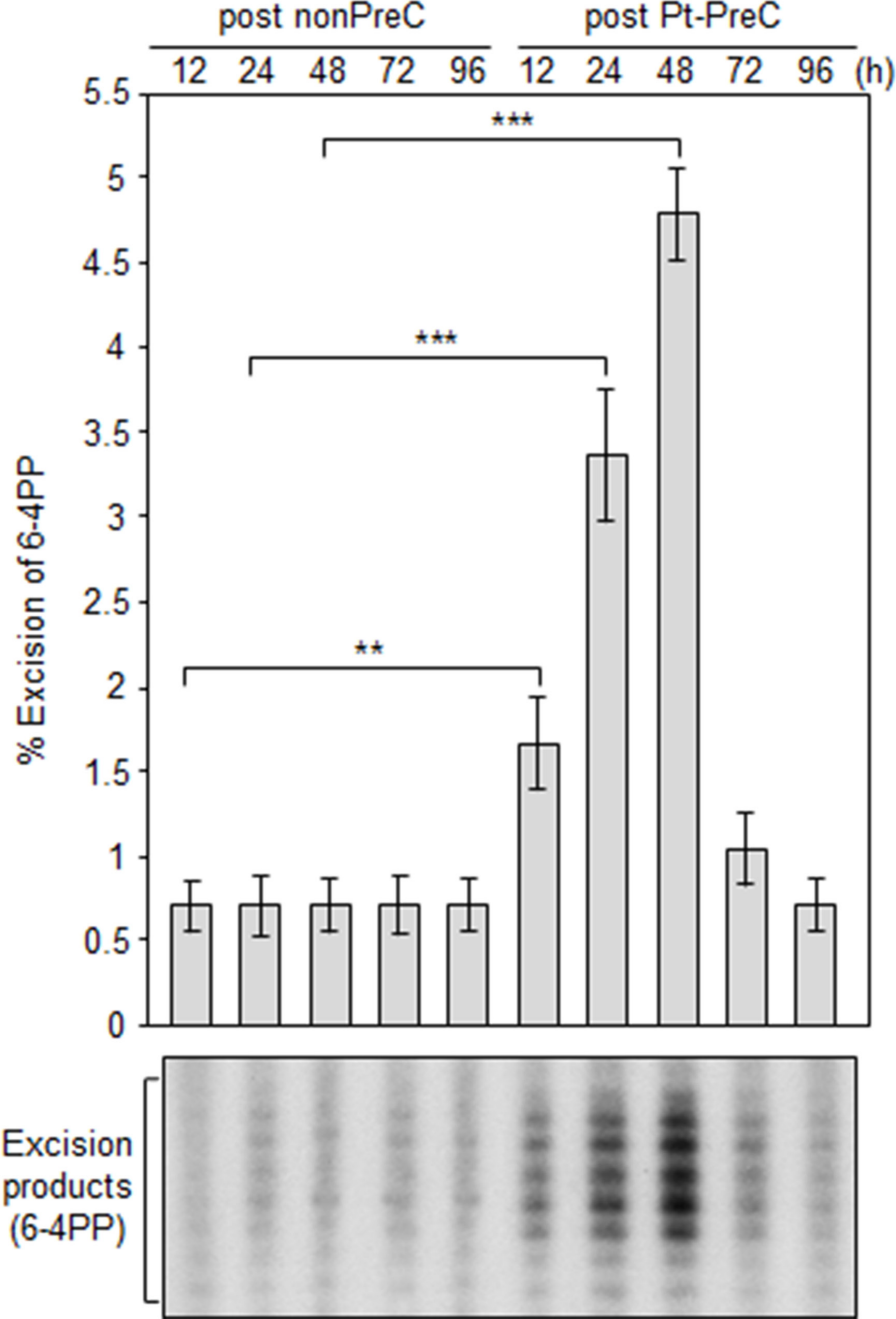
Pt-PreC enhances NER capacity for 6-4 photoproduct (6-4PP) removal Dual-incision NER activity assay was performed using isotope-labeled and 6-4PP-containing linear substrate DNA and cell lysates obtained from nonPreC and Pt-PreC cells at the indicated times after PreC. Amount of excision product was used as a measure of the NER capacity of the lysate. Results are presented as mean ± SD from three independent experiments. Differences were considered significant at the values of *P* < 0.01 (**) and *P* < 0.001 (***).

In order to obtain insight into the effect of DNA repair capacity on the mechanism of chemoresistance, we investigated changes in NER activity after treatment of cells with nonlethal doses of DNA-damaging agents. We used two monoclonal antibodies to specifically detect UV-induced CPDs and Pt-GpG adducts, lesions that are the exclusive substrates of NER [3, 4]. To exclude the effect of cell cycle on DNA repair activity, human non-small cell lung carcinoma A549 and large cell lung carcinoma H460 cells were grown to confluence and kept for additional four days to completely block cell proliferation (Figure 1A–1C) before treatment with DNA damaging agents. Several UV doses were applied to measure the repair activity and cell viability. The amount of CPD lesions on genomic DNA was analyzed by immunoslot blotting (Figure 1D), and cell viability after 24 h of UV exposure was assessed by a fluorescence-based cell viability assay (Figure 1E). Upon irradiation with 5 J/m^2^ UV, there was no significant decrease of cell number and 24 h was sufficient for complete repair of CPDs. However, irradiation of cells with more than 5 J/m^2^, including 10 or 20 J/m^2^, resulted in substantial decrease in cell number, and CPDs still remained on genomic DNA at 24 h after UV irradiation. Based on these results, we chose 5 J/m^2^ of UV dose as a repairable and nonlethal condition for activation of DNA damage response in lung cancer cells, which behaved like recurrent cancer or chemoresistant cells after primary chemotherapy with DNA-damaging agents. We termed this condition as “preconditioning” (PreC) and examined whether it affected subsequent DNA repair activity evoked by cisplatin treatment at a lethal concentration of 10 μM. As shown in Figure 1F and 1G, UV-PreC facilitated repair of subsequent Pt-GpG adduct compared to the nonpreconditioned control (nonPreC). In the inverse experiment, we preconditioned cells with a nonlethal concentration of cisplatin (5 μM) and then investigated repair of the UV-induced CPDs. As expected, repair of CPDs caused by 10 J/m^2^ UV required more time than those induced by 5 J/m^2^ UV (Figure 2A, lanes 1 and 2). This pattern was also observed when cells were preconditioned with cisplatin (Figure 2A, lanes 4 and 5). However, the kinetics of CPD repair after the same dose of UV were much faster when cells had been preconditioned with cisplatin (Figure 2B and 2C), which is similar to the effect of UV-PreC shown in Figure 1. Next, we measured Pt-GpG removal rate following Pt-PreC. As shown in Figure 3A, there was no remaining Pt-GpG adduct after 48 h of Pt-PreC with 5 μM of cisplatin. Meanwhile the repair kinetics upon following cisplatin treatment was much faster in Pt-PreC than nonPreC cells (Figure 3B). These results suggest that NER activity may be upregulated by PreC with a nonlethal dose of DNA-damaging agent. To test this hypothesis we measured the cell's NER capacity at specific times from 12 h to 96 h after PreC. To this end, we used an *in vitro* dual-incision assay, for which we prepared DNA substrate containing UV-induced 6-4PP, which is a better substrate than CPD, and cell lysate prepared at various time points after PreC. Figure 4 shows that the lysate of nonPreC cells had no time-dependent effect on dual-incision activity, whereas the lysate of Pt-PreC cells showed changes in NER capacity depending on the duration of time after PreC. NER activity started to increase 12 h after PreC and peaked at 48 h, at which time the lesions were completely repaired. However the enhancement of NER capacity by PreC was no longer detected 72 h after PreC (Figure 4).

**Figure 5:**
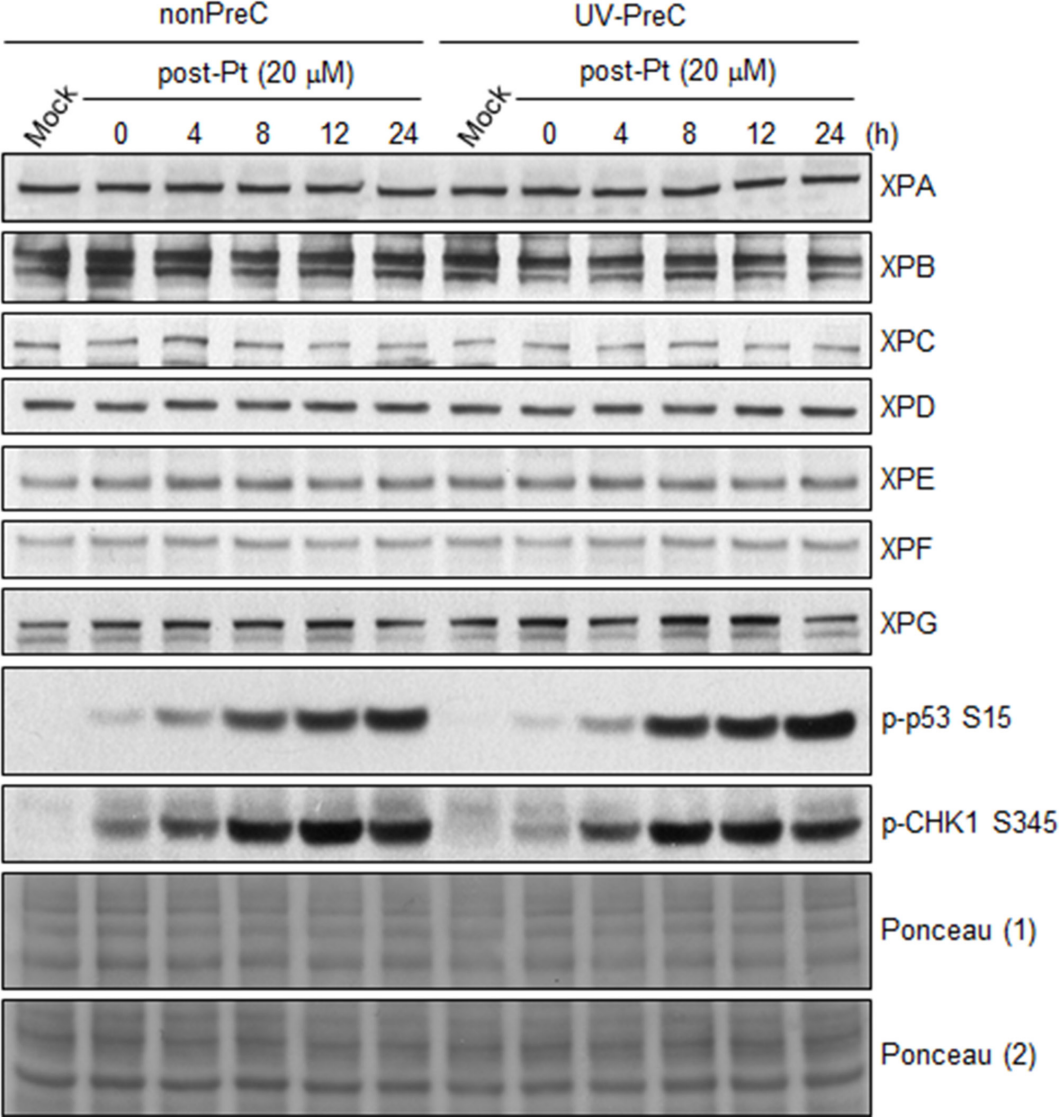
PreC does not alter the protein expression of core NER factors nor ATR activity 24 h later of UV-PreC with 5 J/m^2^, cells were treated with 20 μM of cisplatin for 2 h and then cells were allowed to recover for the indicated times. Protein levels of core NER factors (XPA-XPG) and ATR substrate proteins (p-p53 and p-CHK1) were assessed by immunoblotting with the indicated antibodies. Ponceau stained blots from two different gels were used to indicate equal loading of the samples.

To decipher the mechanism underlying enhancement of NER capacity by PreC, we first analyzed the levels of core NER factors XPA through XPG at 24 h after PreC and compared this with levels in nonpreconditioned controls because some previous reports demonstrated increase of core NER factors including XPA and XPF during adaptive response. As shown in Figure 5, however, there was no significant change in the expression of NER factors regardless of PreC. Next, we analyzed ATR kinase activity indirectly by monitoring the level of phosphorylation of its substrate proteins p53 and CHK1. ATR is known to augment NER activity by phosphorylating and, thus stabilizing, XPA in response to DNA damage [18]. The result indicates that UV-PreC had no effect on ATR activity as no significant alteration in phosphorylation of p53 or CHK1 was detected after PreC (Figure 5). In addition, similar phosphorylation profiles were obtained from nonPreC and UV-PreC cells, which implies that ATR activity had not been altered by PreC.

**Figure 6:**
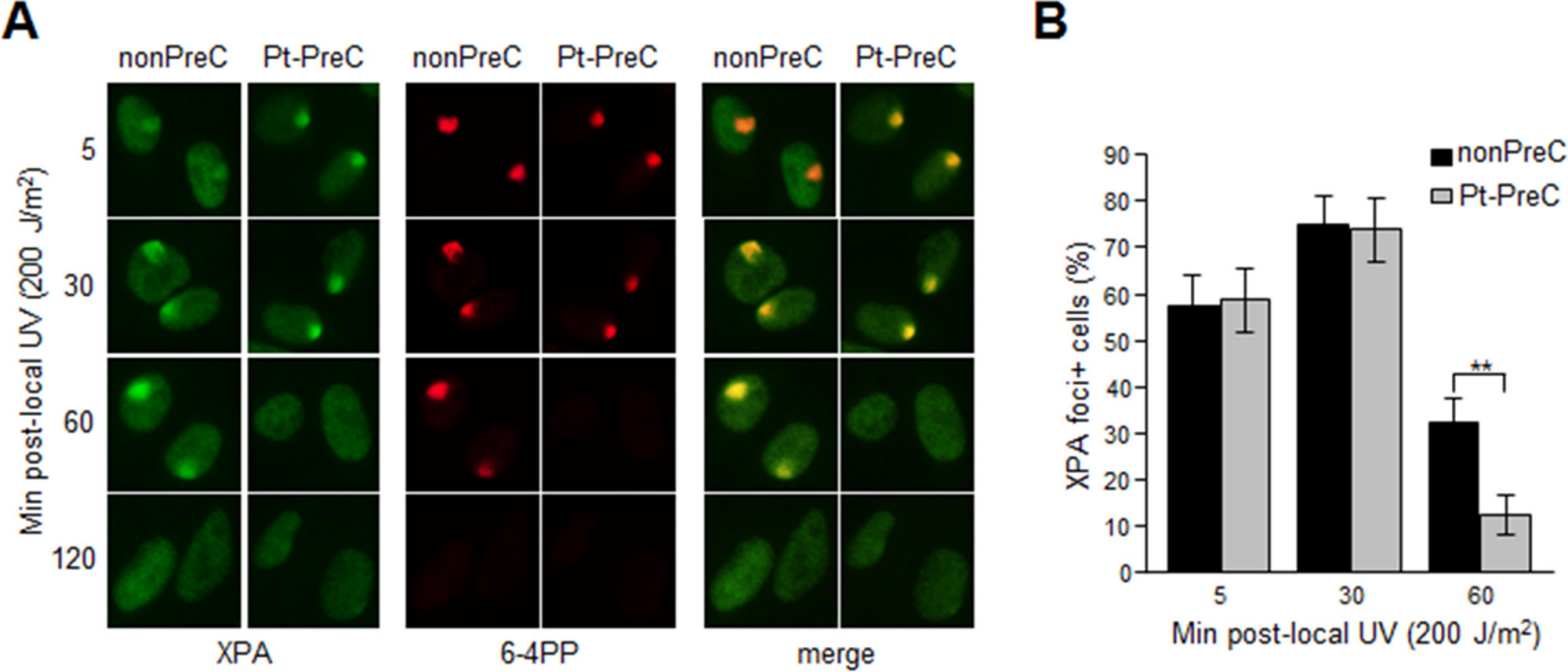
PreC accelerates XPA binding to DNA lesions **A.** 6-4PP removal kinetics and lesion-specific XPA binding from cells preconditioned with nonPreC control or Pt-PreC were monitored after 200 J/m^2^ of local UV irradiation using isopore filter with 5 μm in diameter. **B.** The number of XPA foci-positive cells was calculated among 1000 cells counted. Results are presented as mean ± SD from three independent experiments. Differences were considered significant at the value of *P* < 0.01 (**).

**Figure 7:**
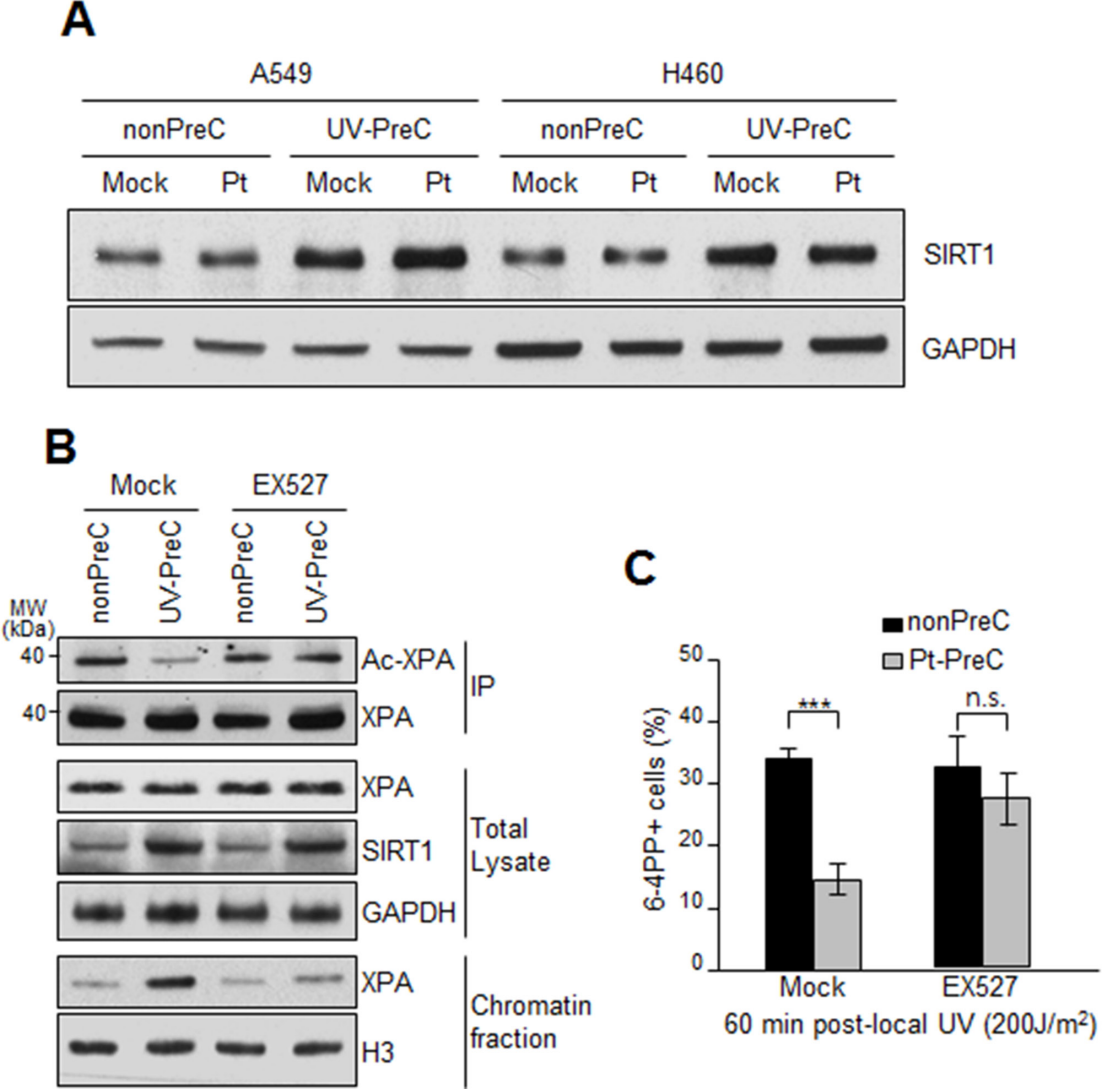
Upregulation of SIRT1 expression during PreC **A.** SIRT1 expression from A549 and H460 preconditioned with nonPreC or UV-PreC was assessed by immunoblotting. GAPDH was used as a loading control. **B.** Acetylation of XPA was assessed by immunoprecipitation of XPA followed by immunoblotting with anti-acetyl-lysine antibody in the presence or absence of EX527, the SIRT1-specific inhibitor. PreC cells were pretreated with EX527 for 5 h before cisplatin treatment. Histone H3 was used to indicate chromatin-enriched fraction. **C.** 6-4PP repair kinetics from nonPreC or Pt-PreC was measured in the presence or absence of EX527. 60 minutes after local UV irradiation the 6-4PP foci-positive cells were counted from randomly selected 1000 cells in each sample. The bars and error bars represent the mean ± s.d from three independent experiments. Differences were considered significant at the value of *P* < 0.001 (***).

XPA is the key rate-limiting factor for NER [11, 14]. However, given that PreC had no effect on XPA protein level or ATR activity, we next examined the effect of PreC on XPA mobility to damaged DNA using local UV irradiation. XPA foci at locally-exposed sites strongly coincided with 6-4PP lesions (Figure 6A). As similar as shown in immunoslot blot data in previous figures, Pt-PreC accelerated the 6-4PP removal than nonPreC control, as demonstrated by more rapid disappearance of 6-4PP signal (Figure 6A). For a quantitative analysis we counted the number of XPA foci-positive cells and found no difference between nonPreC and Pt-PreC within 30 min of recovery time (Figure 6B). However, at 60 min after UV exposure approximately 3 times less XPA foci-positive cells were detected in Pt-PreC, indicating that the Pt-PreC may modulate the efficient XPA recruitment on DNA lesions followed by a robust repair and possibly conferring resistance to toxic DNA damage. Because SIRT1, a histone deacetylase, has been implicated recently in the NER pathway by virtue of deacetylating XPA and thus enhancing NER activity, we measured the level of SIRT1. Interestingly, UV-PreC cells showed increased levels of SIRT1 compared to nonPreC control (Figure 7A). To verify the acetylation status of XPA we immunoprecipitated XPA and determined the acetylation level with anti-acetyl-lysine antibody. The result indeed indicated a decrease in acetylation level of XPA with UV-PreC, which is immediately reversed by treatment of SIRT1 inhibitor EX527 (Figure 7B). To confirm the role of SIRT1 in the PreC effect, we pretreated cells with the specific SIRT1 inhibitor EX-527 [23] before treatment of cisplatin following the UV-PreC and investigated XPA loading to chromatin. UV-PreC-induced enhancement of XPA chromatin loading was reduced in the presence of EX527, implying that the SIRT1 regulated XPA acetyl status may contribute XPA sensitivity to DNA lesions. The PreC-induced repair capacity was also compromised by SIRT1 inhibition (Figure 7C), which implies that upregulation of SIRT1 is the major mechanism in PreC-induced NER potentiation.

## DISCUSSION

An ultimate goal in cancer therapy is to devise individually tailored treatment plans that target growth-promoting pathways and circumvent drug resistance. In general, tumor cells acquire resistance by manipulating biochemical mechanisms that reduce pharmacokinetics or by acquiring additional alterations in DNA damage response pathways. Hence, an understanding of these processes is important for predicting treatment response and for the development of novel treatment strategies for chemoresistance. Most chemotherapeutics rely for their anticancer activity on induction of a DNA damage response to promote the apoptotic pathway [24, 25]. However, DNA repair pathways counteract this effect by repairing damaged DNA and restoring it to normal status. Cisplatin, carboplatin, and oxaliplatin are platinum-based drugs for treatment of many types of cancers, including head and neck, testicular, ovarian, cervical, lung, colorectal, and relapsed lymphoma [3–5, 7]. The cytotoxicity of platinating agents is thought to be due to the platinum intrastrand crosslink that forms on DNA, such as Pt-GpG adduct [3, 4]. Resistance can be caused by a number of cellular adaptations, including reduced uptake, inactivation by intracellular antioxidants, and increased DNA repair capacity [3, 7]. In this report, we identified a plausible mechanism of chemoresistance by which NER capacity is enhanced by preconditioning of cells with a nonlethal dose of DNA-damaging agents (PreC). By virtue of lesion-specific antibodies we were able to precisely measure the repair kinetics caused by specific DNA damage. Pt-GpG adduct removal following UV-PreC, UV-CDP or UV-6-4PP removal following Pt-PreC and Pt-GpG removal following Pt-PreC were enhanced compared to nonPreC control, which led us the general conclusion that PreC makes cells more resistant to subsequent toxic DNA damage at least in lung carcinoma A549 and H460 cells. Some earlier studies had already shown the adaptive response evoked by preconditioning of which are mostly driven by increase of core NER factor expression. For instance, preconditioning cells with a low dose of ionizing radiation (IR) enhances UV-induced NER [26]. In response to IR, transcription of NER genes, such as XPC and DDB2 (XPE), was upregulated due to the stabilization of p53, which induces transcription of the genes. However, in this study we did not detect such an increase in any core NER factors; nor did we detect p53 activation. Instead we found a novel pathway for an adaptive response mediated by upregulation of SIRT1. Because the expression level of XPA, the rate limiting factor in NER was not changed by PreC we assumed that posttranslational modification of XPA occurred during PreC, but that was not mediated by ATR because ATR activity was not altered by PreC. Importantly, we found that increased expression of SIRT1 upon PreC compared to nonPreC control. Reportedly SIRT1 contributed to the decrease of acetylated XPA or increase of deacetylated XPA [20] that showed preferential binding to the DNA damage, required for the enhanced NER capacity induced by PreC. Antagonizing SIRT1 activity using inhibitors such as EX527, sirtinol, and the tenovins has been demonstrated to induce p53-mediated apoptosis [27–29], suggesting that SIRT1 inhibition may be beneficial for treating certain types of cancer. Here we support the SIRT1's role in chemoresistance in some types of lung cancer cells and demonstrate that using SIRT1 inhibitor to treat a certain type of lung cancer showing acquired NER capacity would be beneficial for cancer treatment.

## MATERIALS AND METHODS

### Cell culture and cell viability assay

A549 and H460 cells (American Type Culture Collection, Manassas, VA, USA) were cultured in Dulbecco's modified Eagle medium supplemented with 10% fetal bovine serum and 1% penicillin-streptomycin. 100% confluent cells were kept for additional four days to completely block cell proliferation and then exposed to the UV light using a germicidal lamp (for immnoslot blotting) or UV crosslinker (for immunofluorescence) emitting primarily UV-C light. A UV-C sensor (UV Products, Upland, CA, USA) was used to calibrate the fluence rate of the incident light. For immunofluorescence staining, cells were grown on a glass coverslip coated with poly-D-lysine and laminin (BD Biosciences, San Jose, CA, USA). For assessment of cell viability, CellTiter-Fluor Cell Viability Assay kit (Promega, Madison, WI, USA) was used as indicated in manufacturer's protocol. Fluorescence plate reader (BioRad, Hercules, CA, USA) was used to measure a constitutive protease activity within live cells using a fluorogenic and cell permeable peptide substrate.

### Immunoslot blotting

Genomic DNA was obtained using a QIAamp DNA Mini Kit (Qiagen, Hilden, Germany), and 100 μg (for CPD) or 500 μg (for 6-4PP and platinum [Pt]-GpG adduct) DNA was vacuum-transferred to a nitrocellulose membrane using a BioDot SF Microfiltration apparatus (BioRad). DNA was crosslinked to the membrane by incubation at 80°C for 2 h under vacuum. Monoclonal antibodies that recognize CPD (Kamiya, Seattle, WA, USA), 6-4PP (Cosmo Bio, Tokyo, Japan) and Pt-GpG (Oncolyze, Essen, Germany) were used to detect the amounts of remaining lesions in the genomic DNA. After the immunoslot blot assay, the total DNA amounts loaded onto the membrane were visualized with SYBR-gold staining, and these values were used for normalization.

### Dual-incision NER activity assay

Assay of NER activity in the cell lysate toward 6-4PP-containing DNA substrate was carried out as reported previously [30]. Briefly, 10 fmol of 140-bp duplex with a 6-4PP in the center and ^32^P-label at the 5th phosphodiester bond 5′ to the site of the lesion was incubated with 70 μg of lysate in 25 μL of excision buffer at 30°C for 1 h. The amount of excision product was used as a measure of NER capacity in the lysate. The 6-4PP-containing linear duplex substrate DNA and NER-competent cell lysate were prepared as described previously [16].

### Immunoblotting and immunoprecipitation

Whole-cell lysate prepared as described [31] was used to determine the levels of proteins. Antibodies used in this study include those against XPA (Kamiya), XPB-XPD (Santa Cruz Biotechnology, Santa Cruz, CA, USA), XPE, p-p53, p-CHK1, GAPDH, SIRT1, acetyl-lysine (Cell Signaling Technology), XPF, and XPG (both Abcam, Cambridge, UK). For immunoprecipitation of XPA, 1 mg of whole-cell lysate was incubated with 1 μg of anti-XPA conjugated to Protein A/G-agarose beads (Sigma, St. Louis, MO, USA) for 12 h at 4°C with rotation. After washing with lysis buffer, proteins were eluted from the beads by boiling in SDS sample buffer and resolved on 10% SDS-polyacrylamide gels. For detection of XPA acetylation, anti-acetyl-lysine was employed.

### Local UV irradiation and immunofluorescence

Cells preconditioned with cisplatin were irradiated with UV-C at a dose of 200 J/m^2^ through an isopore polycarbonate filter with pores 5 μm in diameter (EMD Millipore). After platinum preconditioning (Pt-PreC), if necessary, cells were treated with 1 μM of specific SIRT1 inhibitor EX-527 (Sigma) for 5 h before local UV irradiation. After incubation for the recovery times the cells were fixed in 4% paraformaldehyde for 15 min, followed by conventional immunofluorescence staining procedures. UV-induced lesions were counter-labeled with anti-6-4PP antibody, and XPA foci-positive cells were counted for quantitative analysis. The images were captured using Nikon imaging software NIS-Elements 4.0.

### Statistics

Data were evaluated using Student's *t*-test, one-way ANOVA with Tukey test, or two-way ANOVA for multiple comparisons as indicated. Results are presented as mean ± SD from at least three independent experiments. Differences were considered significant at the values of *P* < 0.05 (*), *P* < 0.01 (**), and *P* < 0.001 (***). Statistical analyses were performed with GraphPad Prism 5.0 software (GraphPad, La Jolla, CA, USA).
